# Pomegranate and Its Components as Alternative Treatment for Prostate Cancer

**DOI:** 10.3390/ijms150914949

**Published:** 2014-08-25

**Authors:** Lei Wang, Manuela Martins-Green

**Affiliations:** Department of Cell Biology and Neuroscience, University of California Riverside, Riverside, CA 92521, USA; E-Mail: lwang017@ucr.edu

**Keywords:** natural products, metastasis, luteolin, ellagic acid, punicic acid

## Abstract

Prostate cancer is the second leading cause of cancer deaths in men in the United States. There is a major need for less toxic but yet effective therapies to treat prostate cancer. Pomegranate fruit from the tree *Punica granatum* has been used for centuries for medicinal purposes and is described as “nature’s power fruit”. Recent research has shown that pomegranate juice (PJ) and/or pomegranate extracts (PE) significantly inhibit the growth of prostate cancer cells in culture. In preclinical murine models, PJ and/or PE inhibit growth and angiogenesis of prostate tumors. More recently, we have shown that three components of PJ, luteolin, ellagic acid and punicic acid together, have similar inhibitory effects on prostate cancer growth, angiogenesis and metastasis. Results from clinical trials are also promising. PJ and/or PE significantly prolonged the prostate specific antigen (PSA) doubling time in patients with prostate cancer. In this review we discuss data on the effects of PJ and PE on prostate cancer. We also discuss the effects of specific components of the pomegranate fruit and how they have been used to study the mechanisms involved in prostate cancer progression and their potential to be used in deterring prostate cancer metastasis.

## 1. Introduction

**Figure 1:**
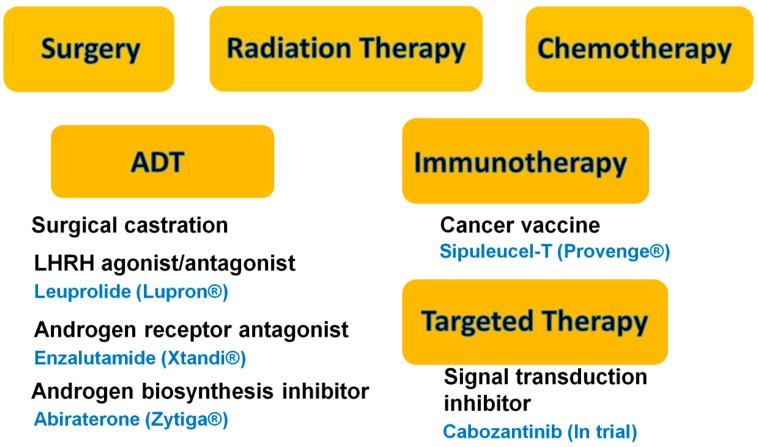
Summary of current treatments against prostate cancer. The current treatments include surgery, radiation, chemotherapy, androgen-deprivation therapy (ADT), immunotherapy, and targeted therapy.

Prostate cancer (PCa) is the second-leading cause of cancer-related deaths in men in the United States. The American Cancer Society has estimated that a total of 233,000 new cases will be diagnosed and 29,480 men will die of PCa in 2014 [1]. Various treatments are available, some more effective than others. To date there is no real cure for the disease beyond surgery and/or radiation when used at early stages of the disease. When recurrence occurs, the cancer can be controlled with hormone ablation therapy (Leuprolide/Lupron^®^), taking advantage of the growth dependence of PCa on testosterone. However, over time, the cancer develops ways to bypass hormone dependence, becoming highly aggressive, castration-resistant prostate cancer (CRPC) that metastasizes to the lung, liver and bone [2,3]. In addition to the hormone ablation, chemotherapy is available today to treat CRPC, but it is not very effective because PCa cells divide slowly and, like with prostatectomy, the treatments are aggressive and have many side effects [4]. As a result, researchers are looking for novel strategies to treat PCa. FDA approved sipuleucel-T (Provenge^®^) is an autologous cellular immunotherapy to treat metastatic PCa. In the clinical trial on which this approval was granted, the median overall survival rate of patients who received sipuleucel-T improved by only 4.5 months. Treatment is costly but some patients survived much longer than the median [5]. Novel androgen receptor (AR) antagonists such as enzalutamide (Xtandi^®^) and androgen biosynthesis inhibitors such as abiraterone (Zytiga^®^), have shown great promise as androgen deprivation therapies to prolong overall survival rate among patients with metastatic PCa [6,7]. Another novel drug, Cabozantinib, is a potent dual inhibitor of the tyrosine kinases c-MET and vascular endothelial growth factor receptor 2 (VEGFR2), and has been shown to reduce or stabilize metastatic bone lesions in CRPC patients [8,9]. These treatments against PCa are summarized in Figure 1. However, all of these treatments have adverse side effects.

More recently, there has been a renewed push to identify natural remedies to fight prostate cancer. Among the latter is pomegranate juice (PJ) and/or pomegranate extracts (PE). The pomegranate fruit is derived from the tree *Punica granatum*, is edible and is cultivated in Mediterranean countries, Afghanistan, India, China, Japan, Russia, and some parts of the United States [10,11]. Pomegranates have been used in folk medicine for centuries. They possess strong antioxidant, anti-inflammatory, anti-atherogenic effects, and some studies have suggested that they also may have anti-tumorigenic properties [12,13,14,15]. In fact, the antioxidant activity of pomegranates has been shown to be higher than that of red wine or green tea, two dietary substances that are showing promise in preclinical prostate cancer models and in patients with PCa [16]. In this review we discuss data on the effects of PJ and PE on PCa cells in cell culture, in animal models and in clinical trials as well as specific components of the pomegranate fruit and how they have been used to study the mechanisms involved in prostate cancer progression.

## 2. Pomegranate Juice (PJ) and/or Pomegranate Extracts (PE) Inhibit Prostate Cancer (PCa) Cell Growth

### 2.1. In Vitro Evidence

A considerable amount of evidence has shown that PJ and/or PE are capable of suppressing the growth of human PCa cell lines *in vitro*. It has been shown that different anatomically discrete fractions of PE induced cell death of three well-characterized PCa cell lines, LNCaP, PC3, and DU 145 [17]. Each of these cell types has specific advantages as a model. LNCaP has functional androgen receptors and hence it is androgen sensitive and secretes prostate-specific antigen (PSA); PC3 cells are androgen independent and are highly invasive with strong metastatic potential; DU145 cells are also androgen independent and highly proliferative but with moderate metastatic capability. These investigators showed that various concentrations of PE (20–100 µg/mL) inhibited proliferation, invasion through Matrigel and induced apoptosis of LNCaP, PC3 and DU145 cells [17]. These findings suggest an overall significant anti-proliferative and pro-apoptotic action of PE against human PCa.

Shortly after these studies were performed, it was found that PE possesses anti-proliferative and pro-apoptotic effects through modulation of cyclin-dependent kinase (cdk) and the cdk inhibitor machinery in PC3 cells [18]. The authors demonstrated that PE inhibited PC3 cell growth by disrupting the cell cycle regulatory molecules in the G1-phase of the cell cycle. It is well-established that cell cycle progression is regulated by the cyclin and cdk complexes. Cyclins D and E are known to regulate cell cycle progression from G1 to S phase [19]. During the progression of the cell cycle, the cdk–cyclin complexes are inhibited via binding to cdk inhibitors such as the p21 and p27 proteins [20]. These investigators showed that PE significantly down-regulated cyclin D1, D2 and E and cdk2, cdk4 and cdk6 and up-regulated p21 and p27, which may cause a blockage of G1–S phase transition, resulting in a G1-phase arrest and apoptosis [18,21]. Furthermore, apoptosis associated proteins such as cleaved poly(ADP-ribose) polymerase (PARP) and Bcl-2-associated X protein (Bax) were also found to be up-regulated in PC3 cells by PE whereas apoptosis blocking proteins such as B-cell lymphoma 2 (Bcl-2) were down-regulated. Bcl-2 is an upstream effector molecule in the apoptotic pathway and is identified as a potent suppressor of apoptosis. Bcl-2 has been shown to form a heterodimer complex with the pro-apoptotic member Bax, rendering it inactive [22]. Therefore, the ratio of Bax to Bcl-2 is a decisive factor and plays an important role in determining whether cells will undergo death or survival. In PE treated cells, the ratio of Bax to Bcl-2 was altered in favor of apoptosis [18,21]. We showed that 1% or 5% PJ inhibited the growth of PC3, DU145 and LNCaP cells in a dose-dependent manner [23]. Collectively, these results suggest that PE inhibits the growth of PCa cells through cell cycle arrest and stimulation of apoptosis.

Because androgen and the androgen receptor play central roles for PCa cell growth and progression, the antiandrogenic effects of PE were studied. It was shown that PE reduces the expression level of androgen biosynthesis genes such as the 3β-hydroxysteroid dehydrogenase type II (HSD3B2) and steroid 5α reductase type I (SRD5A1) genes in LNCaP cells [24]. More recently, PE was shown to reduce the production of testosterone and dihydrotestosterone (DHT) in LNCaP and 22RV1 cells [25]. Therefore, PE may have chemopreventive as well as chemotherapeutic effects against PCa in humans.

### 2.2. In Vivo Evidence

Promising evidence also come from *in vivo* studies using mouse xenograft tumor models. In these studies, human PCa cells were transplanted, either subcutaneously or orthotopically, into immunocompromised mice that do not reject human cells. Tumors were developed and the responses to PE and/or PJ treatment were studied. A single subcutaneous administration of PE (2 µg/g of body weight) before PC3 xenograft tumor implantation in nude mice significantly inhibited tumor growth [17]. Similarly, oral consumption of PE (0.1% and 0.2%, *w*/*v*) inhibited androgen-sensitive CWR22Rv1 xenograft tumor growth in nude mice [18] and also inhibited androgen-independent LAPC4 xenograft tumor growth in severe combined immunodeficient (SCID) mice [26]. Mouse LAPC4 xenograft tumors are initially androgen-dependent and respond to castration but subsequently become androgen-independent after several weeks [27]. These investigators found that PE (0.8 mg/dose/animal) delayed the regrowth of LAPC4 androgen-independent xenograft tumors after castration. In addition, they observed that increases in NF-κB activity during the transition from androgen-dependent to androgen-independent in LAPC4 xenograft tumors were abrogated by PE [26,28].

**Figure 2:**
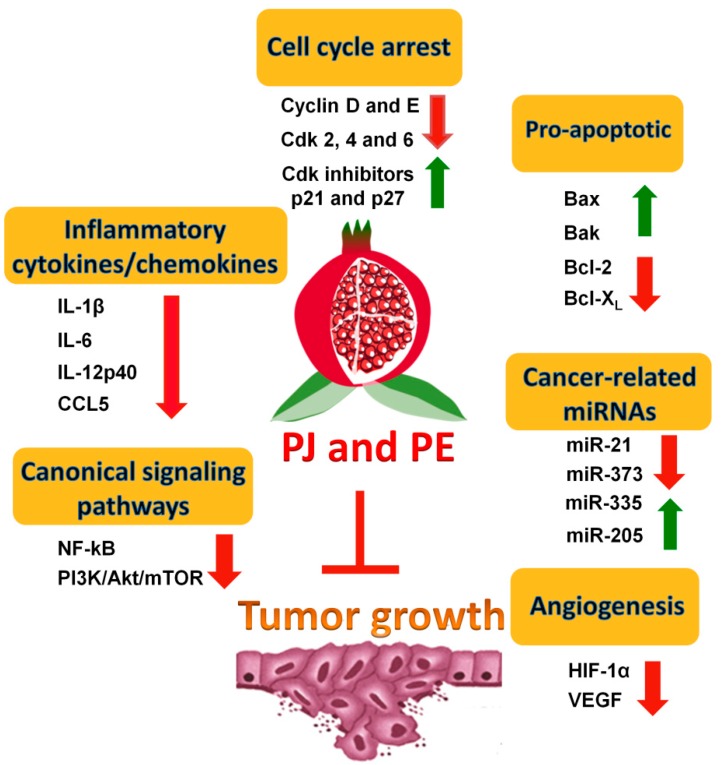
Possible mechanisms of the effects of pomegranate on tumor growth. Possible mechanisms include inducing cell cycle arrest and apoptosis, affecting the level of cancer-related miRNAs, decreasing the level of pro-inflammatory cytokines/chemokines, targeting the canonical signaling pathways, and inhibiting angiogenesis.

To evaluate the chemopreventive effects of PE and/or PJ in preclinical animal models, transgenic mouse models that more closely mimic human prostate cancer progression than xenograft models have been used. Another advantage of transgenic models is that mice are immunocompetent, hence the tumor microenvironment can be more accurately simulated. It has recently been found that PE inhibits tumor growth in a transgenic adenocarcinoma mouse prostate (TRAMP) model [29]. TRAMP mice were fed orally with two doses of PE, equivalent to 250 and 500 mL of PJ, and examined for tumor growth [29]. These investigators found that PE not only inhibited tumor growth but also enhanced the overall survival of the treated mice. The possible mechanisms by which PE could exert its effects include inhibition of PI3K/Akt/mTOR signaling pathways. These findings suggest that PE and/or PJ inhibit PCa development and progression, possibly via targeting this pathway. Possible mechanisms of the effects of pomegranate on PCa tumor growth are summarized in Figure 2. Taken together, the evidence described above establishes a strong potential for PE and/or PJ as chemopreventive agents against PCa.

## 3. Effects of PE and/or PJ on the Metastatic Potential of PCa

### 3.1. Inhibition of the AA Eicosanoid Pathways

**Figure 3:**
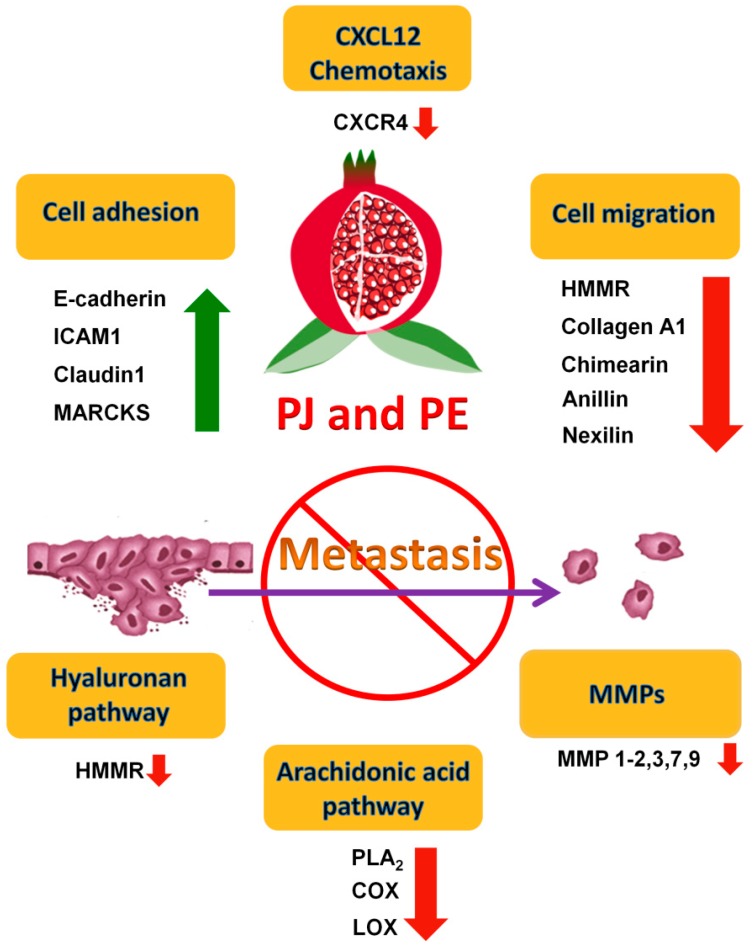
Possible mechanisms of the anti-metastatic effects of pomegranate. Possible mechanisms include increasing cell adhesion, decreasing cell migration and C–X–C motif chemokine 12 (CXCL12) chemotaxis, targeting hyaluronan and arachidonic acid pathway, and reducing matrix metalloproteinases (MMPs) production.

Several studies have shown that the invasive capabilities of metastatic cell lines are significantly reduced following PE and/or PJ treatment (Figure 3). The invasiveness was assessed by Matrigel invasion assay that calculates the percentage of PCa cells that are capable of migrating through the Matrigel membrane after PE and/or PJ treatment [17,30]. It has previously been shown that arachidonic acid (AA) turnover can be an indicator of invasiveness and metastasis [31]. The rate of AA turnover in prostate tumor cells is 10-fold higher than its rate in the surrounding normal cells [32]. AA is released from membrane phospholipids by phospholipase A2 (PLA_2_) and is metabolized into prostaglandins and thromboxanes via the cyclooxygenase (COX) system [33]. Products of COX pathways that are secreted by tumor and/or host cells are thought to influence a variety of biological processes such as cell proliferation, cell movement, carcinogenesis, tumor promotion, and tumor metastasis [34,35,36]. The main product of AA metabolism in PCa cells is PGE_2_ [37]. This prostaglandin has been shown to promote tumor progression by stimulating cancer cell survival and invasiveness through the activation of the PI3K/Akt pathway [38]. Also, the expression of cytosolic PLA_2_ was increased in androgen-independent PCa cells [39,40]. In addition, inhibitors which selectively inhibit PLA_2_ or COX in the prostaglandin synthesis pathway were capable of inhibiting human PCa cell invasion [41]. These results suggest that AA metabolism plays a role in human PCa cell invasion and implicates the involvement of PLA_2_ and COX in a specific invasion associated signal cascade. Furthermore, it has been shown that PE significantly reduced the expression level of PLA_2_ in PC3 cells [30]. Taken together these findings suggest that PE may suppress PCa cell invasion through blocking the AA metabolism pathway.

### 3.2. Inhibition of Matrix Metalloproteinases (MMPs)

The association of matrix proteolysis and cancer has long been demonstrated, and destruction of the basement membrane, an ECM structure that underlies epithelia, is a well-established hallmark of malignant invasion [42,43] (Figure 3). MMPs are a family of highly homologous Zn^2+^ endopeptidases that collectively cleave extracellular matrix (ECM) molecules [44]. Numerous studies have supported a role for MMPs in tumor invasion and metastasis [45,46,47]. MMPs-2, -7, -9 and -11 have all been shown to contribute to tumor progression in studies using MMP-deficient mice [48]. Other studies have shown that MMP-9 was exclusively expressed by malignant prostate tissue and, in particular, by tumors that exhibit aggressive and metastatic phenotypes [49,50]. It has also been shown that inhibitors of both PLA_2_ and COX were able to reduce the production of MMP-2 from PCa cells, indicating that the anti-invasive effect of blocking AA metabolism through the COX pathway is associated with the inhibition of MMPs in tumor progression [41]. PE and/or PJ inhibited the ultraviolet (UV)-induced production of MMP-1, -2, -3, -7 and -9 in reconstituted human skin [51] and inhibited the expression of MMP-1, -3, and -13 in human chondrocytes *in vitro* [52]. Therefore, these studies suggest that the observed anti-invasive effect of PE and/or PJ might involve, at least in part, the reduction of MMP production by PCa cells.

Hyaluronan (HA), also known as hyaluronic acid or hyaluronate, is over-produced by many types of tumor and HA levels are prognostic for malignant progression [53,54,55] (Figure 3). HA is frequently localized in the stroma of solid tumors, facilitating cell migration, tumor invasion and metastasis [56,57]. HA interacts with cell surfaces through binding to specific cell-surface receptors, such as hyaluronan-mediated motility receptor (HMMR), to induce the transduction of a range of intracellular signals leading to numerous cellular responses, including those that involve tyrosine kinases, protein kinase C, focal adhesion kinase (FAK), phosphatidylinositol 3-kinase (PI3K), mitogen-activated protein kinase, nuclear factor-κB and RAS, as well as cytoskeletal components [58]. Previous studies have revealed that the expression level of HMMR was highly stimulated in CRPC cells [59,60,61]. A study using microarray analysis of gene profiles in prostate epithelium obtained from 50 PCa patients showed that major components of the HMMR signaling pathway, such as RhoA-activated kinase (ROK), were consistently overexpressed in metastatic PCa cells [60]. Recently, we showed that PJ or a combination of its components luteolin (L), ellagic acid (E) and punicic acid (P) decreased cell migration, which is an important cellular process in metastasis of PC3 cells, and the inhibitory effect was mediated via down-regulating the expression of HMMR [23,62]. Therefore, these findings suggest that the anti-metastatic effect of PJ and of L + E + P also occur via targeting HA signaling pathways in PCa cells.

### 3.3. Inhibition of Pro-Inflammatory Cytokines and Chemokines

It is known that, with time, cancer cells develop ways to bypass the need for testosterone and then cancer progresses very rapidly. Approximately 80% of patients who have died of advanced CRPC have clinical evidence of bone metastases and 100% have histologic bone involvement [63,64]. It has been shown that the CXCL12/CXCR4 axis may play a critical role in the metastasis of prostate cancer to lung, liver and bone [65,66,67,68]. Other studies showed that activated CXCL12/CXCR4 axis not only greatly increased the invasiveness but also induced the production of MMPs in PCa cells [69]. We have recently shown that PJ inhibits chemotaxis towards CXCL12 in DU145, PC3 and LNCaP cells [23,62]. These findings show that targeting the CXCL12/CXCR4 axis in PCa cells by L + E + P, leads to inhibition of metastasis.

Recently, we showed that, in addition to causing cell death of androgen-independent PCa cells, PJ also increases cell adhesion and decreases cell migration, two important cellular processes involved in metastasis [23]. PJ up-regulates genes involved in cell adhesion such as *E*-cadherin, intercellular adhesion molecule 1 (ICAM1) and myristoylated alanine-rich protein kinase C (MARCKS) and down-regulates genes involved in cell migration such as type I collagen, tenascin C and chimerin 1. In addition, anti-invasive microRNAs such as miR-335 (predicted targets include *COL1A1*, *TNC*, *SOX4*), miR-205 (predicted targets include *CHN1*, *PRKCE*), miR-200 (predicted targets include *ZEB1*, *ZEB2*), and miR-126 (predicted targets include *SLC45A3*), are up-regulated, whereas pro-invasive microRNA such as miR-21 (predicted targets include *MARCKS*, *PDCD4*, *TPM1*) and miR-373 (predicted targets include *CD44*), are down-regulated. Moreover, we found that PJ significantly reduces the level of secreted pro-inflammatory cytokines/chemokines such as IL-6, IL-12p40, IL-1β and RANTES, thereby having the potential for decreasing inflammation and its impact on cancer progression [70,71,72,73]. The possible mechanisms of the anti-metastatic effects of pomegranate are summarized in Figure 3. Taken together, these findings strongly suggest the potential of pomegranate as a chemopreventive agent to inhibit metastasis in humans.

## 4. Specific Components of Pomegranate with Known Effects on PCa

The pomegranate fruit can be divided into three major anatomical components: the juice, the pericarp and the seeds. These discrete components of the pomegranate fruit have been found to exert anti-proliferative and anti-invasive effects on PCa cells [30]. The juice and pericarp contain a rich complement of two types of polyphenolic components which have attracted interest for recent research: anthocyanins which give the juice its red color [74], such as delphinidin, cyanidin and pelargonidin, and hydrolyzable tannins, such as the punicalagin and gallagic acid [13,75,76]. Other polyphenolic components of possible interest include kaempferol, quercetin and luteolin [75,77,78]. The seed oil, which is comprised of 65%–80% conjugated fatty acids, also contains many compounds of interest with known antioxidant and anti-cancer activities [79]. The predominant component among these fatty acids is punicic acid (present at about 1–5 µg/mL in juice) [80,81].

PJ is a very complex mixture of components and is found in many different formulations, it is important to identify specific components that can replace the effects of the juice on growth and metastasis. Ellagitannin, the most abundant polyphenol present in PJ, is hydrolyzed to ellagic acid (present at about 50–200 µg/mL in juice) that is then converted to urolithin A by gut microflora [82,83]. Oral administration of ellagitannin-enriched PE not only inhibited LAPC4 xenograft tumor growth in SCID mice [84] but also inhibited tumor-associated angiogenesis [85]. In addition, ellagitannin inhibited the expression of androgen receptor (AR) and androgen synthesizing enzymes, such as 3β-hydroxysteroid dehydrogenase type 2 (HSD3B2) and steroid 5α reductase type I (SRD5A1), in PCa cells [24]. Ellagic acid was shown to possess anti-tumorigenic activities on lung, cervical, colon, breast and prostate cancer cells [86,87,88]. Recently it was shown that ellagic acid and its metabolite urolithin A synergistically inhibited cell growth and induced apoptosis in DU145 and PC3 cells [89]. These investigators also found that ellagic acid is more effective than urolithin A.

Several studies have shown anti-proliferative effects of luteolin (present at about 1 µg/mL in juice) on human squamous liver and colon cancer cells [90,91,92]. Using a Matrigel invasion assay, it was shown that luteolin inhibits invasion of PC3 cells via increasing the expression of *E*-cadherin [93]. It is established that decreased expression of *E*-cadherin, one important cell-cell adhesion molecule [94,95], results in a loss of cell-cell adhesion and increased cell invasion [96]. It was found that luteolin increased the expression of *E*-cadherin, and that knockdown of *E*-cadherin reversed the effect of luteolin on invasion of PC3 cells; in addition, intraperitoneal administration of luteolin three times a week reduced lung metastasis of PC3 xenograft tumors in nude mice [93]. In addition, luteolin has been shown to inhibit PCa tumor growth via targeting angiogenesis. After primary xenograft PC3 tumors have developed in nude mice, it was found that intraperitoneal administration of luteolin for 16 days not only inhibited tumor growth, but also reduced the number of blood vessels in the tumor [97].

Punicic acid, the major component of pomegranate seed oil (70%–80%), has been shown to possess anti-cancer effects on PCa. It inhibits cell growth of androgen-dependent LNCaP cells stimulated by DHT and inhibited DHT-stimulated androgen receptor nuclear accumulation as well as the expression of androgen receptor-dependent genes [81]. These investigators also found that punicic acid induced apoptosis via a caspase-dependent pathway in LNCaP cells.

Recently, we showed that luteolin (L), ellagic acid (E) and punicic acid (P), individually and in combination, additively affect processes important for metastasis. L + E + P in equal amounts inhibits the growth of hormone-dependent and -independent PCa cells, their migration and their chemotaxis towards CXCL12, a factor that is important in PCa metastasis [62]. The combination of these components also increases the expression of cell adhesion genes and decreases expression of genes involved in cell cycle control and cell migration. Furthermore, we found that L + E + P increases several well-known anti-invasive miRNAs, such as miR-200c and miR-335, while decreasing several oncogenic miRNAs, such as miR-21 and miR-29b. We have also shown similar anti-metastatic effects of L + E + P on breast cancer cells [98].

**Figure 4:**
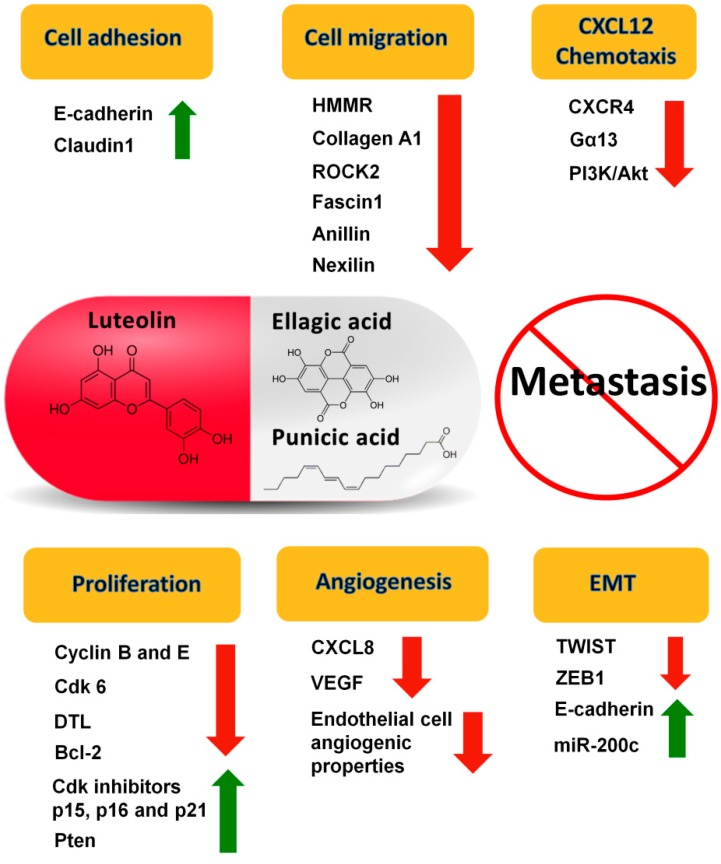
Possible mechanisms of the anti-metastatic effects of the combination of luteolin (L), ellagic acid (E) and punicic acid (P) (L + E + P). Possible mechanisms include increasing cell adhesion, decreasing cell migration and CXCL12 chemotaxis, inhibiting proliferation, inhibiting angiogenesis, and inhibiting epithelial-mesenchymal transition (EMT).

Based on these results in cell culture, we investigated whether L + E + P inhibits PCa metastasis *in vivo*. We used a SCID mouse model in which luciferase-expressing human PCa cells were injected ectopically in the region of the prostate. One advantage of this model is that tumor growth and metastasis can be monitored by bioluminescence imaging. We found that L + E + P significantly inhibited PC-3M-luc primary tumor growth and that none of the tumors treated with L + E + P metastasized. One disadvantage of using this model to study metastasis is the relative low metastasis incidence in untreated mice. However, it has been shown that inhibition of the PTEN/PI3K pathway combined with activation of the Ras/MAPK pathway promotes prostate cancer metastasis [99]. Therefore, we further tested the effects of L + E + P on highly invasive Pten^−/−^; K-ras^G12D^ PCa cells and found that L + E + P not only inhibited tumor growth but also inhibited lung and liver metastasis. L + E + P also significantly inhibited the CXCL12/CXCR4 axis for metastasis *in vivo*, consistent with our findings *in vitro*. In addition, we found that L + E + P inhibited angiogenesis in PC-3M-luc and Pten^−/−^; K-ras^G12D^ tumors. We also showed that L + E + P decreased human endothelial cell migration and adhesion, disrupted endothelial tube formation and inhibited the production of the angiogenic factors IL-8 or VEGF by the tumors [100]. The possible mechanisms of the anti-metastatic effects of L + E + P are summarized in Figure 4. These results show that L + E + P can inhibit PCa progression/metastasis, indicating potential use of this combination of natural products for treatment of PCa in humans.

## 5. Clinical Trials of PE and/or PJ on PCa

To investigate the effects of PJ consumption on PCa progression in men, a phase II clinical trial for men with rising PSA after surgery or radiotherapy was conducted in 2006 [101]. Patients were treated with 8 ounces of PJ daily until disease progression. During the trial, there were no serious adverse events reported and the treatment was well tolerated. Mean PSA doubling time significantly increased with treatment from a mean of 15 months at baseline to 54 months post-treatment (*p* < 0.001). The investigators also found that PJ treatment suppressed cell proliferation and increased apoptosis in the prostate cancer cell line LNCaP. In addition, PJ treatment increased serum nitric oxide and reduced the oxidative state and sensitivity to oxidation of serum lipids in patients. No patients developed metastases during the period of the trial period.

In 2013, another phase II clinical trial of PCa patients with rising PSA received 1 g (comparable to about 8 oz of PJ) or 3 g of PE daily for up to 18 months. PSA doubling time lengthened more than 6 months from 11.9 to 18.5 months (*p* < 0.001) with no significant difference between dose groups [102]. Again, no patient developed metastases during the trial period. The statistically significant prolongation of PSA doubling time and the lack of metastatic progression in any of the patients in both of these studies, strongly suggests the potential of PJ for treatment of PCa.

Although extremely promising, a major drawback of these two clinical trials was the absence of a proper placebo control group. A recent randomized double blind study showed that men with PCa prior to radical prostatectomy were given PE daily for up to 4 weeks. No serious side effects were observed and the treatment was well tolerated. The level of 8-hydroxy-2-deoxyguanosine (8-OHdG), an oxidative stress biomarker, was compared between patients in treatment and placebo group. PE lowered the level of 8-OHdG but the difference was not statistically significant, suggesting the need for larger and longer studies in the future [103].

More recently, results from a double-blind, placebo-controlled randomized trial using an oral capsule containing a blend of pomegranate, green tea, broccoli and turmeric showed that the supplement delayed PCa progression as indicated by slower increases in PSA levels. The median increase in PSA levels in the supplement group was 14.7% as compared to 78.5% in placebo group [104], suggesting the great potential of the food supplement to prevent PCa progression.

## 6. Conclusions

In summary, the biological activity of pomegranate-derived products, especially the chemotherapeutic and chemopreventive properties, has been investigated in cell, animal and clinical studies. The findings discussed in this review show that pomegranate and its components interfere with multiple biological processes involved in tumor growth, angiogenesis and metastasis of PCa. Because many of the molecular mechanisms are shared by different types of cancers, and the fact that PE has been shown to be effective against breast, lung, colon and skin cancer [105] further enhances the therapeutic potential of PE. Therefore, further studies are warranted, including clinical trials with appropriate control groups using well-characterized and standardized amounts of PJ, PE and specific components as primary or adjuvant therapy in men with PCa. Many of the molecular mechanisms involved in the PJ/PE or L + E + P are amenable to drug treatment and to the development of small inhibitory molecules and therefore allow for combination therapy. Therefore, pomegranate and its components can potentially be used to prevent development and progression of PCa as well as other cancers. What is not known and is of great importance is whether the power of PJ/PE or L + E + P can be used as preventive therapies.

## References

[1] American Cancer Society http://www.cancer.org/.

[2] Stavridi F., Karapanagiotou E.M., Syrigos K.N. (2010). Targeted therapeutic approaches for hormone-refractory prostate cancer. Cancer Treat. Rev..

[3] Chuu C.P., Kokontis J.M., Hiipakka R.A., Fukuchi J., Lin H.P., Lin C.Y., Huo C., Su L.C. (2011). Androgens as therapy for androgen receptor-positive castration-resistant prostate cancer. J. Biomed. Sci..

[4] Hoffman-Censits J., Fu M. (2013). Chemotherapy and targeted therapies: Are we making progress in castrate-resistant prostate cancer?. Semin. Oncol..

[5] Higano C.S., Small E.J., Schellhammer P., Yasothan U., Gubernick S., Kirkpatrick P., Kantoff P.W. (2010). Sipuleucel-T. Nat. Rev. Drug Discov..

[6] Sartor O., Pal S.K. (2013). Abiraterone and its place in the treatment of metastatic crpc. Nat. Rev. Clin. Oncol..

[7] Ryan C.J., Smith M.R., de Bono J.S., Molina A., Logothetis C.J., de Souza P., Fizazi K., Mainwaring P., Piulats J.M., Ng S. (2013). Abiraterone in metastatic prostate cancer without previous chemotherapy. N. Engl. J. Med..

[8] Yakes F.M., Chen J., Tan J., Yamaguchi K., Shi Y., Yu P., Qian F., Chu F., Bentzien F., Cancilla B. (2011). Cabozantinib (xl184), a novel met and vegfr2 inhibitor, simultaneously suppresses metastasis, angiogenesis, and tumor growth. Mol. Cancer Ther..

[9] Smith D.C., Smith M.R., Sweeney C., Elfiky A.A., Logothetis C., Corn P.G., Vogelzang N.J., Small E.J., Harzstark A.L., Gordon M.S. (2013). Cabozantinib in patients with advanced prostate cancer: Results of a phase II randomized discontinuation trial. J. Clin. Oncol..

[10] Longtin R. (2003). The pomegranate: Nature’s power fruit?. J. Natl. Cancer Inst..

[11] Langley P. (2000). Why a pomegranate?. BMJ.

[12] Gil M.I., Tomas-Barberan F.A., Hess-Pierce B., Holcroft D.M., Kader A.A. (2000). Antioxidant activity of pomegranate juice and its relationship with phenolic composition and processing. J. Agric. Food Chem..

[13] Lansky E.P., Newman R.A. (2007). *Punica granatum* (pomegranate) and its potential for prevention and treatment of inflammation and cancer. J. Ethnopharmacol..

[14] Kim N.D., Mehta R., Yu W., Neeman I., Livney T., Amichay A., Poirier D., Nicholls P., Kirby A., Jiang W. (2002). Chemopreventive and adjuvant therapeutic potential of pomegranate (*Punica granatum*) for human breast cancer. Breast Cancer Res. Treat..

[15] Khan N., Afaq F., Mukhtar H. (2008). Cancer chemoprevention through dietary antioxidants: Progress and promise. Antioxid. Redox Signal..

[16] Noda Y., Kaneyuki T., Mori A., Packer L. (2002). Antioxidant activities of pomegranate fruit extract and its anthocyanidins: Delphinidin, cyanidin, and pelargonidin. J. Agric. Food Chem..

[17] Albrecht M., Jiang W., Kumi-Diaka J., Lansky E.P., Gommersall L.M., Patel A., Mansel R.E., Neeman I., Geldof A.A., Campbell M.J. (2004). Pomegranate extracts potently suppress proliferation, xenograft growth, and invasion of human prostate cancer cells. J. Med. Food.

[18] Malik A., Afaq F., Sarfaraz S., Adhami V.M., Syed D.N., Mukhtar H. (2005). Pomegranate fruit juice for chemoprevention and chemotherapy of prostate cancer. Proc. Natl. Acad. Sci. USA.

[19] Sanchez I., Dynlacht B.D. (2005). New insights into cyclins, cdks, and cell cycle control. Semin. Cell Dev. Biol..

[20] Harper J.W., Adami G.R., Wei N., Keyomarsi K., Elledge S.J. (1993). The p21 cdk-interacting protein cip1 is a potent inhibitor of g1 cyclin-dependent kinases. Cell.

[21] Malik A., Mukhtar H. (2006). Prostate cancer prevention through pomegranate fruit. Cell Cycle.

[22] Oltersdorf T., Elmore S.W., Shoemaker A.R., Armstrong R.C., Augeri D.J., Belli B.A., Bruncko M., Deckwerth T.L., Dinges J., Hajduk P.J. (2005). An inhibitor of bcl-2 family proteins induces regression of solid tumours. Nature.

[23] Wang L., Alcon A., Yuan H., Ho J., Li Q.J., Martins-Green M. (2011). Cellular and molecular mechanisms of pomegranate juice-induced anti-metastatic effect on prostate cancer cells. Integr. Biol..

[24] Hong M.Y., Seeram N.P., Heber D. (2008). Pomegranate polyphenols down-regulate expression of androgen-synthesizing genes in human prostate cancer cells overexpressing the androgen receptor. J. Nutr. Biochem..

[25] Ming D.S., Pham S., Deb S., Chin M.Y., Kharmate G., Adomat H., Beheshti E.H., Locke J., Guns E.T. (2014). Pomegranate extracts impact the androgen biosynthesis pathways in prostate cancer models *in vitro* and *in vivo*. J. Steroid Biochem. Mol. Biol..

[26] Rettig M.B., Heber D., An J., Seeram N.P., Rao J.Y., Liu H., Klatte T., Belldegrun A., Moro A., Henning S.M. (2008). Pomegranate extract inhibits androgen-independent prostate cancer growth through a nuclear factor-κB-dependent mechanism. Mol. Cancer Ther..

[27] Klein K.A., Reiter R.E., Redula J., Moradi H., Zhu X.L., Brothman A.R., Lamb D.J., Marcelli M., Belldegrun A., Witte O.N. (1997). Progression of metastatic human prostate cancer to androgen independence in immunodeficient SCID mice. Nat. Med..

[28] McCarty M.F., Hejazi J., Rastmanesh R. (2014). Beyond androgen deprivation: Ancillary integrative strategies for targeting the androgen receptor addiction of prostate cancer. Integr. Cancer Ther..

[29] Adhami V.M., Siddiqui I.A., Syed D.N., Lall R.K., Mukhtar H. (2012). Oral infusion of pomegranate fruit extract inhibits prostate carcinogenesis in the tramp model. Carcinogenesis.

[30] Lansky E.P., Jiang W., Mo H., Bravo L., Froom P., Yu W., Harris N.M., Neeman I., Campbell M.J. (2005). Possible synergistic prostate cancer suppression by anatomically discrete pomegranate fractions. Investig. New Drugs.

[31] Honn K.V., Bockman R.S., Marnett L.J. (1981). Prostaglandins and cancer a review of tumor initiation through tumor metastasis. Prostaglandins.

[32] Chaudry A., McClinton S., Moffat L.E.F., Wahle K.W.J. (1991). Essential fatty acid distribution in the plasma and tissue phospholipids of patients with benign and malignant prostatic disease. Br. J. Cancer.

[33] Hyde C.A., Missailidis S. (2009). Inhibition of arachidonic acid metabolism and its implication on cell proliferation and tumour-angiogenesis. Int. Immunopharmacol..

[34] Piomelli D. (1993). Arachidonic acid in cell signaling. Curr. Opin. Cell Biol..

[35] Schneider C., Pozzi A. (2011). Cyclooxygenases and lipoxygenases in cancer. Cancer Metastasis Rev..

[36] Khan Z., Khan N., Tiwari R.P., Sah N.K., Prasad G.B., Bisen P.S. (2011). Biology of Cox-2: An application in cancer therapeutics. Curr. Drug Targets.

[37] Yang P., Cartwright C.A., Li J., Wen S., Prokhorova I.N., Shureiqi I., Troncoso P., Navone N.M., Newman R.A., Kim J. (2012). Arachidonic acid metabolism in human prostate cancer. Int. J. Oncol..

[38] Sheng H., Shao J., Washington M.K., DuBois R.N. (2001). Prostaglandin E2 increases growth and motility of colorectal carcinoma cells. J. Biol. Chem..

[39] Ghosh J., Myers C.E. (1998). Inhibition of arachidonate 5-lipoxygenase triggers massive apoptosis in human prostate cancer cells. Proc. Natl. Acad. Sci. USA.

[40] Patel M.I., Singh J., Niknami M., Kurek C., Yao M., Lu S., Maclean F., King N.J., Gelb M.H., Scott K.F. (2008). Cytosolic phospholipase A2-α: A potential therapeutic target for prostate cancer. Clin. Cancer Res..

[41] Attiga F.A., Fernandez P.M., Weeraratna A.T., Manyak M.J., Patierno S.R. (2000). Inhibitors of prostaglandin synthesis inhibit human prostate tumor cell invasiveness and reduce the release of matrix metalloproteinases. Cancer Res..

[42] Brinckerhoff C.E., Matrisian L.M. (2002). Matrix metalloproteinases: A tail of a frog that became a prince. Nat. Rev. Mol. Cell Biol..

[43] Kessenbrock K., Plaks V., Werb Z. (2010). Matrix metalloproteinases: Regulators of the tumor microenvironment. Cell.

[44] Page-McCaw A., Ewald A.J., Werb Z. (2007). Matrix metalloproteinases and the regulation of tissue remodelling. Nat. Rev. Mol. Cell Biol..

[45] Sternlicht M.D., Werb Z. (2001). How matrix metalloproteinases regulate cell behavior. Annu. Rev. Cell Dev. Biol..

[46] Friedl P., Wolf K. (2008). Tube travel: The role of proteases in individual and collective cancer cell invasion. Cancer Res..

[47] Overall C.M., Lopez-Otin C. (2002). Strategies for MMP inhibition in cancer: Innovations for the post-trial era. Nat. Rev. Cancer.

[48] Nelson A.R., Fingleton B., Rothenberg M.L., Matrisian L.M. (2000). Matrix metalloproteinases: Biologic activity and clinical implications. J. Clin. Oncol..

[49] Hamdy F.C., Fadlon E.J., Cottam D., Lawry J., Thurrell W., Silcocks P.B., Anderson J.B., Williams J.L., Rees R.C. (1994). Matrix metalloproteinase 9 expression in primary human prostatic adenocarcinoma and benign prostatic hyperplasia. Br. J. Cancer.

[50] Hadler-Olsen E., Winberg J.O., Uhlin-Hansen L. (2013). Matrix metalloproteinases in cancer: Their value as diagnostic and prognostic markers and therapeutic targets. Tumor Biol..

[51] Afaq F., Zaid M.A., Khan N., Dreher M., Mukhtar H. (2009). Protective effect of pomegranate-derived products on UVB-mediated damage in human reconstituted skin. Exp. Dermatol..

[52] Ahmed S., Wang N., Hafeez B.B., Cheruvu V.K., Haqqi T.M. (2005). Punica granatum l. Extract inhibits IL-1β-induced expression of matrix metalloproteinases by inhibiting the activation of map kinases and NF-κB in human chondrocytes *in vitro*. J. Nutr..

[53] Auvinen P., Tammi R., Parkkinen J., Tammi M., Agren U., Johansson R., Hirvikoski P., Eskelinen M., Kosma V.M. (2000). Hyaluronan in peritumoral stroma and malignant cells associates with breast cancer spreading and predicts survival. Am. J. Pathol..

[54] Aaltomaa S., Lipponen P., Tammi R., Tammi M., Viitanen J., Kankkunen J.P., Kosma V.M. (2002). Strong stromal hyaluronan expression is associated with PSA recurrence in local prostate cancer. Urol. Int..

[55] Lipponen P., Aaltomaa S., Tammi R., Tammi M., Agren U., Kosma V.M. (2001). High stromal hyaluronan level is associated with poor differentiation and metastasis in prostate cancer. Eur. J. Cancer.

[56] Turley E.A. (1992). Hyaluronan and cell locomotion. Cancer Metastasis Rev..

[57] Toole B.P., Zoltan-Jones A., Misra S., Ghatak S. (2005). Hyaluronan: A critical component of epithelial-mesenchymal and epithelial-carcinoma transitions. Cells Tissues Organs.

[58] Turley E.A., Noble P.W., Bourguignon L.Y.W. (2002). Signaling properties of hyaluronan receptors. J. Biol. Chem..

[59] Gust K.M., Hofer M.D., Perner S.R., Kim R., Chinnaiyan A.M., Varambally S., Moller P., Rinnab L., Rubin M.A., Greiner J. (2009). Rhamm (CD168) is overexpressed at the protein level and may constitute an immunogenic antigen in advanced prostate cancer disease. Neoplasia.

[60] Lin S.-L., Chang D., Chiang A., Ying S.-Y. (2008). Androgen receptor regulates CD168 expression and signaling in prostate cancer. Carcinogenesis.

[61] Lin S.-L., Chang D., Ying S.-Y. (2007). Hyaluronan stimulates transformation of androgen-independent prostate cancer. Carcinogenesis.

[62] Wang L., Ho J., Glackin C., Martins-Green M. (2012). Specific pomegranate juice components as potential inhibitors of prostate cancer metastasis. Transl. Oncol..

[63] Roudier M.P., Vesselle H., True L.D., Higano C.S., Ott S.M., King S.H., Vessella R.L. (2003). Bone histology at autopsy and matched bone scintigraphy findings in patients with hormone refractory prostate cancer: The effect of bisphosphonate therapy on bone scintigraphy results. Clin. Exp. Metastasis.

[64] Loberg R.D., Gayed B.A., Olson K.B., Pienta K.J. (2005). A paradigm for the treatment of prostate cancer bone metastases based on an understanding of tumor cell-microenvironment interactions. J. Cell. Biochem..

[65] Bubendorf L., Schopfer A., Wagner U., Sauter G., Moch H., Willi N., Gasser T.C., Mihatsch M.J. (2000). Metastatic patterns of prostate cancer: An autopsy study of 1589 patients. Hum. Pathol..

[66] Furusato B., Mohamed A., Uhlen M., Rhim J.S. (2010). CXCR4 and cancer. Pathol. Int..

[67] Hirbe A.C., Morgan E.A., Weilbaecher K.N. (2010). The CXCR4/SDF-1 chemokine axis: A potential therapeutic target for bone metastases?. Curr. Pharm. Des..

[68] Wang J., Loberg R., Taichman R.S. (2006). The pivotal role of CXCL12 (SDF-1)/CXCR4 axis in bone metastasis. Cancer Metastasis Rev..

[69] Taichman R.S., Cooper C., Keller E.T., Pienta K.J., Taichman N.S., McCauley L.K. (2002). Use of the stromal cell-derived factor-1/CXCR4 pathway in prostate cancer metastasis to bone. Cancer Res..

[70] Culig Z., Steiner H., Bartsch G., Hobisch A. (2005). Interieukin-6 regulation of prostate cancer cell growth. J. Cell. Biochem..

[71] Cooper A.M., Khader S.A. (2007). IL-12p40: An inherently agonistic cytokine. Trends Immunol..

[72] Apte R.N., Dotan S., Elkabets M., White M.R., Reich E., Carmi Y., Song X., Dvozkin T., Krelin Y., Voronov E. (2006). The involvement of IL-1 in tumorigenesis, tumor invasiveness, metastasis and tumor-host interactions. Cancer Metastasis Rev..

[73] Vaday G.G., Peehl D.M., Kadam P.A., Lawrence D.M. (2006). Expression of CCL5 (rantes) and CCR5 in prostate cancer. Prostate.

[74] Hernandez F., Melgarejo P., Tomas-Barberan F.A., Artes F. (1999). Evolution of juice anthocyanins during ripening of new selected pomegranate (punica granatum) clones. Eur. Food Res. Technol..

[75] Van Elswijk D.A., Schobel U.P., Lansky E.P., Irth H., van der Greef J. (2004). Rapid dereplication of estrogenic compounds in pomegranate (*Punica granatum*) using on-line biochemical detection coupled to mass spectrometry. Phytochemistry.

[76] Gomez-Caravaca A.M., Verardo V., Toselli M., Segura-Carretero A., Fernandez-Gutierrez A., Caboni M.F. (2013). Determination of the major phenolic compounds in pomegranate juices by HPLC–DAD–ESI-MS. J. Agric. Food Chem..

[77] Ackland M.L., van de Waarsenburg S., Jones R. (2005). Synergistic antiproliferative action of the flavonols quercetin and kaempferol in cultured human cancer cell lines. In Vivo.

[78] Qu W., Breksa A.P., Pan Z., Ma H. (2012). Quantitative determination of major polyphenol constituents in pomegranate products. Food Chem..

[79] Schubert S.Y., Lansky E.P., Neeman I. (1999). Antioxidant and eicosanoid enzyme inhibition properties of pomegranate seed oil and fermented juice flavonoids. J. Ethnopharmacol..

[80] Grossmann M.E., Mizuno N.K., Schuster T., Cleary M.P. (2010). Punicic acid is an OMEGA-5 fatty acid capable of inhibiting breast cancer proliferation. Int. J. Oncol..

[81] Gasmi J., Sanderson J.T. (2010). Growth inhibitory, antiandrogenic, and pro-apoptotic effects of punicic acid in lncap human prostate cancer cells. J. Agric. Food Chem..

[82] Seeram N.P., Henning S.M., Zhang Y., Suchard M., Li Z., Heber D. (2006). Pomegranate juice ellagitannin metabolites are present in human plasma and some persist in urine for up to 48 hours. J. Nutr..

[83] Heber D. (2008). Multitargeted therapy of cancer by ellagitannins. Cancer Lett..

[84] Seeram N.P., Aronson W.J., Zhang Y., Henning S.M., Moro A., Lee R.-P., Sartippour N., Harris D.M., Rettig M., Suchard M.A. (2007). Pomegranate ellagitannin-derived metabolites inhibit prostate cancer growth and localize to the mouse prostate gland. J. Agric. Food Chem..

[85] Sartippour M.R., Seeram N.P., Rao J.Y., Moro A., Harris D.M., Henning S.M., Firouzi A., Rettig M.B., Aronson W.J., Pantuck A.J. (2008). Ellagitannin-rich pomegranate extract inhibits angiogenesis in prostate cancer *in vitro* and *in vivo*. Int. J. Oncol..

[86] Castonguay A., Gali H., Perchellet E., Gao X., Boukharta M., Jalbert G., Okuda T., Yoshida T., Hatano T., Perchellet J. (1997). Antitumorigenic and antipromoting activities of ellagic acid, ellagitannins and oligomeric anthocyanin and procyanidin. Int. J. Oncol..

[87] Narayanan B.A., Geoffroy O., Willingham M.C., Re G.G., Nixon D.W. (1999). P53/p21 (WAF1/CIP1) expression and its possible role in G1 arrest and apoptosis in ellagic acid treated cancer cells. Cancer Lett..

[88] Losso J.N., Bansode R.R., Trappey A., Bawadi H.A., Truax R. (2004). *In vitro* anti-proliferative activities of ellagic acid. J. Nutr. Biochem..

[89] Vicinanza R., Zhang Y., Henning S.M., Heber D. (2013). Pomegranate juice metabolites, ellagic acid and urolithin a, synergistically inhibit androgen-independent prostate cancer cell growth via distinct effects on cell cycle control and apoptosis. Evid.-Based Complement. Alternat. Med..

[90] Huang Y.T., Hwang J.J., Lee P.P., Ke F.C., Huang J.H., Huang C.J., Kandaswami C., Middleton E., Lee M.T. (1999). Effects of luteolin and quercetin, inhibitors of tyrosine kinase, on cell growth and metastasis-associated properties in a431 cells overexpressing epidermal growth factor receptor. Br. J. Pharmacol..

[91] Selvendiran K., Koga H., Ueno T., Yoshida T., Maeyama M., Torimura T., Yano H., Kojiro M., Sata M. (2006). Luteolin promotes degradation in signal transducer and activator of transcription 3 in human hepatoma cells: An implication for the antitumor potential of flavonoids. Cancer Res..

[92] Lim Y., Jeong Y., Tyner A.L., Park J.H. (2007). Induction of cell cycle arrest and apoptosis in HT-29 human colon cancer cells by the dietary compound luteolin. Am. J. Physiol. Gastrointest. Liver Physiol..

[93] Zhou Q., Yan B., Hu X., Li X.B., Zhang J., Fang J. (2009). Luteolin inhibits invasion of prostate cancer pc3 cells through *E*-cadherin. Mol. Cancer Ther..

[94] Perez-Moreno M., Jamora C., Fuchs E. (2003). Sticky business: Orchestrating cellular signals at adherens junctions. Cell.

[95] Berx G., van Roy F. (2009). Involvement of members of the cadherin superfamily in cancer. Cold Spring Harb. Perspect. Biol..

[96] Thiery J.P. (2002). Epithelial-mesenchymal transitions in tumour progression. Nat. Rev. Cancer.

[97] Pratheeshkumar P., Son Y.O., Budhraja A., Wang X., Ding S., Wang L., Hitron A., Lee J.C., Kim D., Divya S.P. (2012). Luteolin inhibits human prostate tumor growth by suppressing vascular endothelial growth factor receptor 2-mediated angiogenesis. PLoS One.

[98] Rocha A., Wang L., Penichet M., Martins-Green M. (2012). Pomegranate juice and specific components inhibit cell and molecular processes critical for metastasis of breast cancer. Breast Cancer Res. Treat..

[99] Mulholland D.J., Kobayashi N., Ruscetti M., Zhi A., Tran L.M., Huang J., Gleave M., Wu H. (2012). Pten loss and RAS/MAPK activation cooperate to promote emt and metastasis initiated from prostate cancer stem/progenitor cells. Cancer Res..

[100] Wang L., Garcia M., Mulholland D., Lily M., Martins-Green M. (2014). Luteolin, ellagic acid and punicic acid are natural products that inhibit prostate cancer metastasis. Carcinogenesis.

[101] Pantuck A.J., Leppert J.T., Zomorodian N., Aronson W., Hong J., Barnard R.J., Seeram N., Liker H., Wang H., Elashoff R. (2006). Phase II study of pomegranate juice for men with rising prostate-specific antigen following surgery or radiation for prostate cancer. Clin. Cancer Res..

[102] Paller C.J., Ye X., Wozniak P.J., Gillespie B.K., Sieber P.R., Greengold R.H., Stockton B.R., Hertzman B.L., Efros M.D., Roper R.P. (2013). A randomized phase II study of pomegranate extract for men with rising psa following initial therapy for localized prostate cancer. Prostate Cancer Prostatic Dis..

[103] Freedland S.J., Carducci M.A., Kroeger N., Partin A.W., Rao J., Jin Y., Kerkoutian S., Wu H., Li Y., Creel P. (2013). A double-blind, randomized, neoadjuvant study of the tissue effects of pomx pills in men with prostate cancer prior to radical prostatectomy. Cancer Prev. Res..

[104] Thomas R., Williams M., Sharma H., Chaudry A., Bellamy P. (2014). A double-blind, placebo-controlled randomised trial evaluating the effect of a polyphenol-rich whole food supplement on PSA progression in men with prostate cancer—The U.K. NCRN POMI-T study. Prostate Cancer Prostatic Dis..

[105] Adhami V.M., Khan N., Mukhtar H. (2009). Cancer chemoprevention by pomegranate: Laboratory and clinical evidence. Nutr. Cancer.

